# Effect of alcohol intoxication on the risk of venous thromboembolism: A nationwide retrospective cohort study

**DOI:** 10.1097/MD.0000000000008041

**Published:** 2017-10-20

**Authors:** Chih-Jung Shen, Chia-Hung Kao, Tai-Yi Hsu, Chih-Yu Chen, Cheng-Li Lin, Hong-Mo Shih

**Affiliations:** a Department of Emergency Medicine; b School of Medicine, College of Medicine; c Graduate Institute of Clinical Medical Science and School of Medicine, College of Medicine; d Department of Nuclear Medicine and PET Center; e Department of Bioinformatics and Medical Engineering, Asia University, Taichung, Taiwan Management Office for Health Data; f Management Office for Health Data, China Medical University Hospital, Taichung, Taiwan, Republic of China.

**Keywords:** alcohol intoxication, National Health Insurance Research Database, venous thromboembolism

## Abstract

This study investigated whether alcohol intoxication (AI) increases the risk of venous thromboembolism (VTE) by using the Taiwan National Health Insurance Research Database (NHIRD).

Using data from the NHIRD, we assembled 61,229 patients with acute AI and randomly selected 244,916 controls. Each patient was monitored from 2000 to 2011 to identify those who were subsequently diagnosed with deep vein thrombosis (DVT) and pulmonary embolism (PE). Cox proportional hazard regression analysis was conducted to determine the risk of VTE in the patients with AI compared with the controls.

The incidence rate of DVT during the 10 years follow-up period was 9.36 per 10,000 person-years and 2.07 per 10,000 person-years in the AI and non-AI cohorts, respectively. Moreover, the incidence rate of PE was 4 per 10,000 person-years in the AI cohort and 0.93 in the non-AI cohort. After adjustment for age, sex, and comorbidities, the risks of DVT and PE were 3.40 [95% confidence interval (CI) = 2.83–4.08] and 3.53 (95% CI = 2.69–4.65)-fold higher in the AI cohort than in the non-AI cohort.

An increased incidence of VTE was observed among patients with AI. Therefore, physicians should carefully estimate the risk of VTE in patients with AI.

## 1 Introduction

Venous thromboembolism (VTE) is a major health problem. Deep vein thrombosis (DVT) is the formation of blood clots in one or more deep veins. Acute pulmonary thromboembolism (PE), a severe and sometimes fatal complication of VTE, is the sudden occlusion of a pulmonary artery by a blood clot. In the United States, the average annual incidence of VTE among the White population is 108 per 100,000 person-years.^[1]^ Immobilization, postoperation, trauma, oral contraceptives, cancer, and acute medical illness are believed to provoke the risk factors of VTEs.^[2]^

Alcohol is believed to have a protective effect on cardiovascular disease. A meta-analysis by Ronksley et al^[3]^ demonstrated that light to moderate alcohol consumption is associated with a reduced risk of multiple cardiovascular outcomes, including coronary heart disease, coronary heart disease mortality, and incidence of stroke.

However, excessive alcohol use can lead to a variety of medical and behavioral problems. Alcohol use has been associated with an increased risk of cancer, with heavy use being associated with the highest risk.^[4]^ In addition, excessive alcohol consumption is associated with hepatic injuries, including alcoholic fatty liver disease, alcoholic hepatitis, and cirrhosis.^[5]^ In contrast to the cardiovascular benefits received from light to moderate alcohol consumption, binge drinking can lead to increased risk and mortality of cardiovascular disease.^[6,7]^ In one study, excessive alcohol consumption accounted for 1 in 10 deaths among working-age adults in the United States.^[8]^ Some studies also have indicated that heavy alcohol consumption increases the risk of all strokes, including ischemic and embolic strokes.^[9,10]^

Atherothrombosis and VTE share common risk factors and the pathophysiological characteristics of inflammation, endothelial injury, and hypercoagulability. However, previous studies had nonconclusive results between alcohol abuse and VTE.^[11–13]^ The enrollment of different beverages and varies alcohol consumption habits may cause the inconsistent results. We assume that episodes of binge drinking can be a risk factor for VTE.^[14]^ People who have been admitted to hospital because of acute alcohol intoxication (AI) may have problems of alcohol abuse and more episodes of binge alcohol drinking.^[15]^ Here, we conducted a population-based retrospective cohort study, using records from the Taiwan National Health Insurance Research Database (NHIRD), to investigate whether people with a history of AI are at risk for VTE.

## 2 Methods

### 2.1 Data source

The NHIRD is a computerized database that contains the claims data of people insured by Taiwan's National Health Insurance (NHI) program. The NHI program is a nationwide insurance program that covers ambulatory care, hospital admissions, dental care, prescription drugs, intervention procedures, and disease profiles for more than 99% of Taiwanese residents.^[12]^ The details of the NHI program have been reported elsewhere in previous studies.^[16,17]^ The disease diagnoses that are used in the NHIRD were coded according to the criteria of the International Classification of Disease, Ninth Revision, Clinical Modification (ICD-9-CM). Personal identification information in the NHIRD is scrambled before being released to protect the privacy of patients and healthcare providers. This study was approved by the Institutional Review Board of China Medical University (CMUH-104-REC2–115).

### 2.2 Sampled participants

We identified patients more than 20-years old with newly diagnosed AI (ICD-9-CM codes 303, 305.0, V113, and A215) from hospitalization records from 2000 to 2011 as the AI cohort. The index date for the patients with AI was the date of their first admission visit. We excluded patients who had received a diagnosis of DVT (ICD-9-CM 453.8) or PE (ICD-9-CM 415.1, excluding ICD-9-CM 415.11) before the index date and participants with missing age- or sex-related information. For each AI patient, 4 non-AI comparisons were randomly selected from the pool of participants without AI, DVT, or PE at the baseline, and frequency matched by the year of index date, age (every 5-years span), and sex. Moreover, we performed a sensitivity analysis by using the propensity score method.^[18]^ We used logistic regression to calculate the propensity score for each patient by estimating the assignment probability based on baseline variables, including age, sex, and comorbidities of hypertension, diabetes, cerebral vascular disease, heart failure, all cancer, pregnancy, atrial fibrillation, lower leg fracture or surgery, and chronic liver disease and cirrhosis. This would provide an equal probability to each AI patient of being assigned to the non- AI cohort.

### 2.3 Outcome and comorbidities

The primary outcomes were newly diagnosed PE or DVT from hospitalization records. All of the participants were followed-up on to evaluate the occurrence of primary outcomes until December 31, 2011 or they were censored because of death, withdrew from the NHI program, or were lost to follow up. The pre-existing comorbidities included hypertension (ICD-9-CM codes 401–405), diabetes (ICD-9-CM 250), cerebrovascular disease (CVA) (ICD-9-CM 430–438), heart failure (ICD-9-CM 428), cancer (ICD-9-CM 140–208), pregnancy (ICD-9-CM procedure 72–74 or ICD-9-CM codes 640.x1–676.x1, 640.x2–676.x2, and 650–659), atrial fibrillation (ICD-9-CM 427.31), lower leg fracture or surgery (ICD-9-CM 820–823 and procedure codes 81.51, 81.52, 81.53, and 81.54), and liver cirrhosis (ICD-9-CM code 571).

### 2.4 Statistical analysis

We compared the differences in demographic characteristics, including age, sex, and comorbidity, between the AI and non-AI cohorts by using a *χ*^*2*^ analysis for the categorical variables and Student *t* tests for the continuous variables. The incidence density rate was calculated as the number of incident DVT or PE cases identified during the follow up, divided by the total person-years of follow up for each cohort according to sex, age, and comorbidity. Univariable and multivariable Cox proportional hazards regression models were used to calculate the hazard ratios (HRs) and 95% confidence intervals (CIs) of the risks of DVT and PE in AI patients, relative to those of the non-AI cohort. When the patients were stratified according to age, sex, and comorbidities, the relative risk of DVT and PE in the AI cohort compared with the non- AI cohort was also analyzed using Cox models (Table 2). We used multiplicative analysis to evaluate the interaction effect of AI and sex, and AI and comorbidities on DVT or PE risk (Table 3). Data management and analysis were performed using SAS software (version 9.4, SAS Institute, Cary, NC). A 2-sided *P* < .05 was considered statistically significant.

**Table 2:**
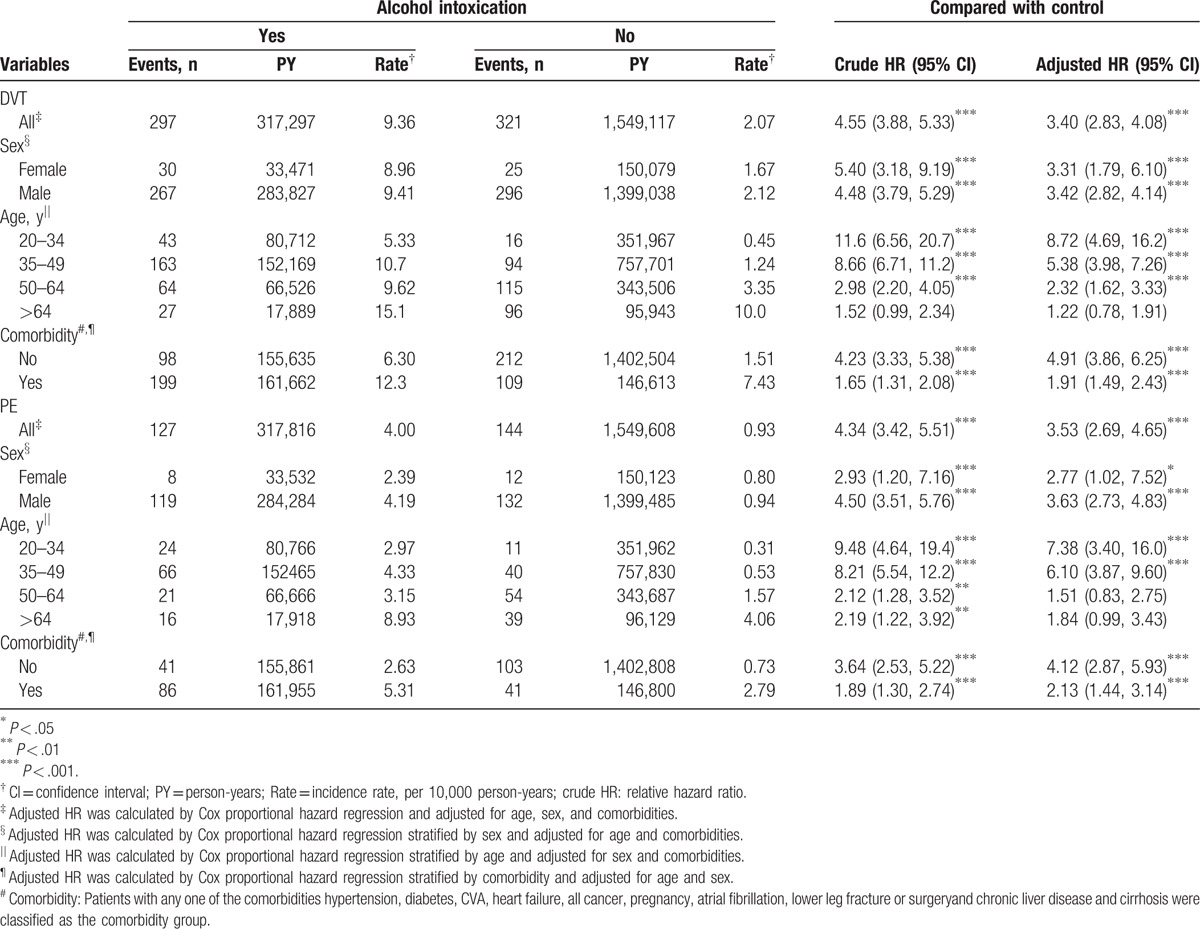
Incidence and adjusted hazard ratio of DVT and PE by sex, age, and comorbidity for alcohol intoxication patients compared with controls.

**Table 3:**
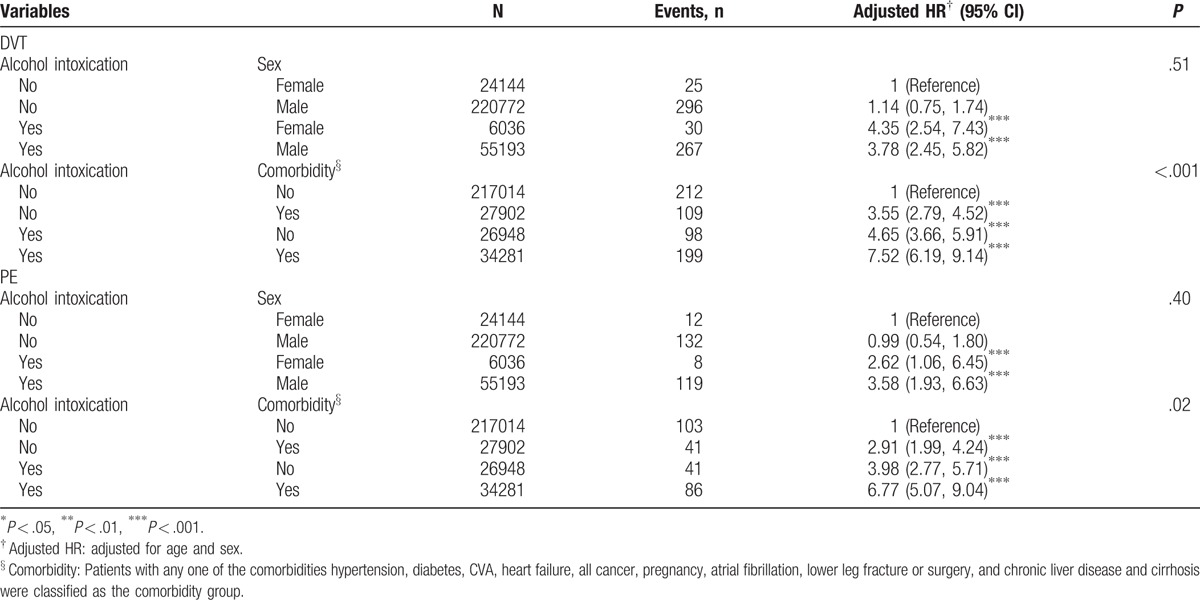
Cox proportional hazard regression analysis for the risk of DVT and PE associated alcohol intoxication with joint effect of sex and comorbidity.

## 3 Results

**Figure 1:**
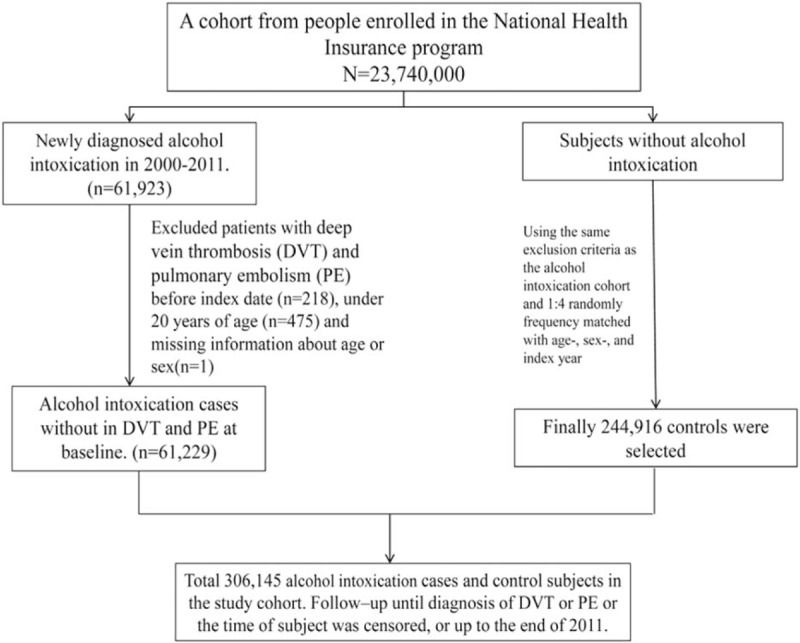
Study flowchart showing the retrieval of participants to form the alcohol intoxication cohort and the control cohort. DVT = deep vein thrombosis, PE = pulmonary embolism.

The eligible study participants consisted of 61,229 patients in the AI cohort and 244,916 individuals in the non-AI cohort. Figure 1 shows the selection procedure of study participants. Most of the participants in this study were men (90.1%), and nearly seven-tenths were younger than 50-years old. The mean ages in the AI and non-AI cohorts were 44.8 ± 12.8 and 44.4 ± 12.9 years, respectively. Most of the patients with AI tended to also have hypertension, diabetes, CVA, heart failure, cancer, pregnancy, atrial fibrillation, lower leg fracture or surgery, and chronic liver disease, and cirrhosis compared with the non-AI cohort (Table 1).

**Table 1:**
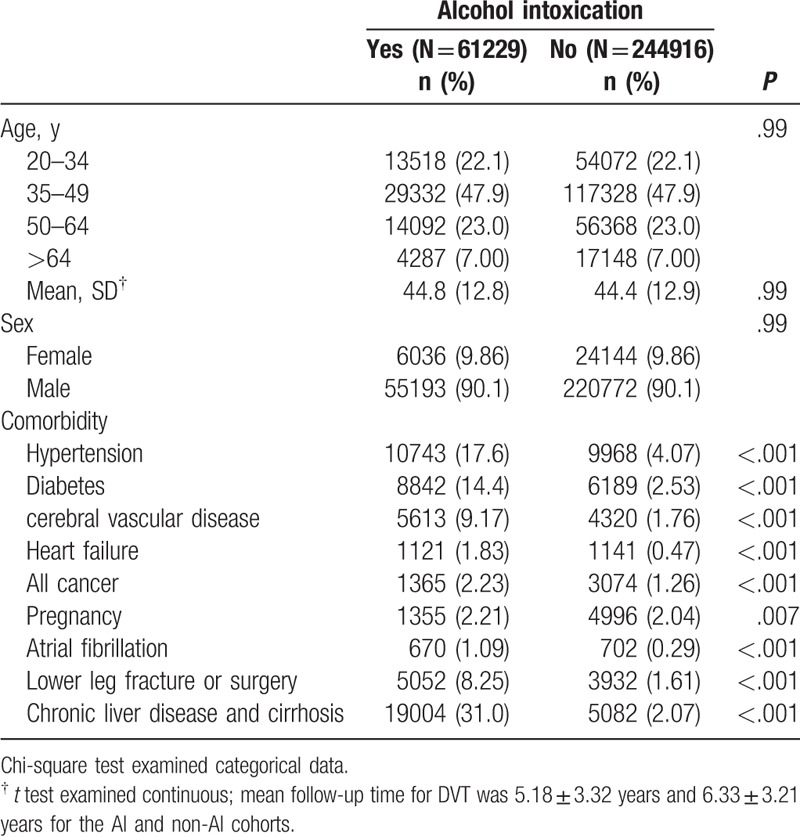
Comparison of demographics and comorbidity between alcohol intoxication patients and controls.

**Figure 2:**
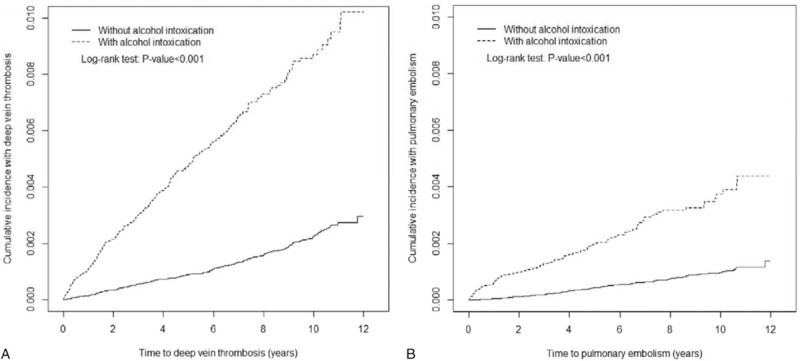
Cummulative incidence of deep vein thrombosis (A) and pulmonary embolism (B) in patients with alcohol intoxication and comparison patients.

The mean follow-up time for DVT was 5.18 ± 3.32 years and 6.33 ± 3.21 years for the AI and non-AI cohorts, respectively. The cumulative DVT incidence curve for the AI cohort revealed a significantly higher incidence of DVT in the AI patients compared with those in the non-AI cohort (log-rank *P* < .001, Fig. 2A). Moreover, compared with the non-AI cohort, the AI cohort exhibited higher incidence rates of DVT (9.36 vs 2.07 per 10 000 person-years), with an adjusted HR (aHR) of 3.40 (95% CI = 2.83–4.08) after adjustment for sex, age, and comorbidities (Table 2). The overall incidence and risk of DVT were compared between the AI and non-AI cohorts in relation to several variables including age, sex, and presence or absence of comorbidity. The risk of DVT in patients with AI at all stratifications was higher than that of the non-AI cohort, except for the older age group (>64-years old).

During the mean follow up for PE at 5.19 years for the AI cohort and 6.33 years for the non-AI cohort, the cumulative incidence of PE in the AI cohort was significantly higher than that in the non-AI cohort (Fig. 2B; *P* < .001). The incidence of PE in patients with AI was 4.00 per 10,000 person-years, whereas the incidence in patients without AI reached 0.93 per 10,000 person-years. After multivariates adjustment, the risk of PE was determined to be 3.53-fold higher in the AI cohort than in the non-AI cohort (95% CI = 2.69–4.65). The sex-specific aHRs of PE for the AI and non-AI cohorts were significant among both women (aHR = 2.77, 95% CI = 1.02–7.52) and men (aHR = 3.63, 95% CI = 2.73–4.83). The incidence density rate of PE increased with age in both cohorts. However, the age-specific aHRs in the AI and non-AI cohorts were higher for the 20 to 34 years (aHR = 7.83, 95% CI = 3.40–16.0) and the 35 to 49 years (aHR = 6.10, 95% CI = 3.87–9.60) age groups. In addition, the patients with AI without comorbidities had a higher risk of PE than the non-AI controls without comorbidities (aHR = 4.12, 95% CI = 2.87–5.93).

The joint effect measures between AI and sex or AI and comorbidity for the risk of DVT and PE are presented in Table 3. Compared with women without AI, women with AI exhibited a higher risk of DVT (aHR = 4.35, 95% CI = 2.54–7.43), followed by men with AI (aHR = 3.78, 95% CI = 2.45, 5.82). Relative to the non-AI cohort without comorbidity, the patients with AI with comorbidity were at a higher risk of DVT (aHR = 7.52, 95% CI = 6.19–9.14). Furthermore, compared with women without AI, men with AI had a higher risk of PE (aHR = 3.58, 95% CI = 1.93–6.63). The risk of PE was also significantly higher among the patients with AI with comorbidity (aHR = 6.77, 95% CI = 5.07–9.04) compared with the participants in the non-AI cohort without comorbidity.

The incidences for DVT in the AI cohort, and the propensity score matched non-AI cohort were 8.34, and 3.69 per 10,000 person-years, respectively (Table 4). AI patients had a 2.49-fold risk of DVT compared with propensity score matched non-AI cohort (95% CI = 1.96–3.15). The patients in the AI cohort had a 2.88-fold higher PE risk than those with propensity score matched non-AI cohort (95% CI = 1.96–4.24).

**Table 4:**
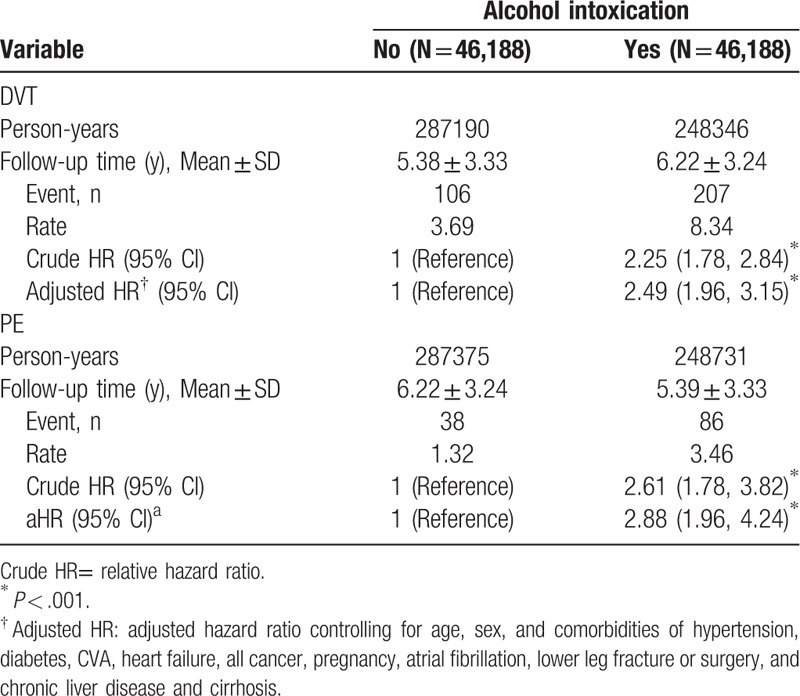
Overall incidence of DVT and PE (per 10,000 person-years) and estimated hazard ratios according to disease status by the propensity score method.

## 4 Discussion

The main result of this population-based retrospective cohort study in Taiwan is that patients with AI are associated with a higher risk of VTE. To best of our knowledge, this is the first study discovered the association between AI and VTE. This finding is consistent with that of the Tromsø study, which indicated that binge drinking is associated with an increased risk of VTE.^[14]^ One recent review by Lippi G et al^[11]^ showed inconsistent results between alcohol abuse and VTE. The nonconclusive results may come from different drinking habits. In this study, we found the AI cohort, who could have more binge drinking episodes, had higher incidence of VTE. Although the patients with AI had a higher proportion of comorbid diseases than those without AI, AI remains an independent risk factor for VTE after multivariate adjustment.

The NHIRD covers nearly 99% of the population of Taiwan and is a representative data source that includes age, sex, and comorbidity information. Because each resident in Taiwan is assigned a unique personal identification number, all of the study participants could be traced through the NHI records for the entire follow-up period. This enabled us to conduct this study, which is the first nationwide study on an Asian population that contains a large sample size as well as a long follow-up period; moreover, our findings can be generalized to the entire population of Taiwan.

Table 2 shows the incidence of VTE with increasing age. The AI cohort exhibited a higher hazard ratio for individuals aged between 20 and 50 years. Although not statistically significant, a higher incidence was observed among those older than 50 years. This result may be because of the increased age, which is a risk factor for VTE, as demonstrated by Oger in 2000.^[19]^ Also the increased comorbidities, such as cancer and stroke, among elder patients could also moderate the incidence of AI. There were increased widths of 95% CIs among young and female subjects. This could be result from the relative small sample size of these 2 groups. We also found that there were much fewer AI events among female subjects. However, the risk of VTEs in women with AI remained higher than non-AI cohort. Non-AI male had a slightly higher incidence of DVT. In contrast, AI female had a higher risk of DVT. Although there were more comorbidities in AI cohort (Table 1), patients with AI without comorbidities had a 4.65-fold higher risk of DVT and 3.98-fold higher risk of PE, which were significantly higher than those of the patients in the control groups (Table 3). To reduced potential bias, we matched controls with propensity score, as showed in Table 4. The AI cohort still had a higher risk of VTEs.

Although our results do not reveal the mechanism for this association, according to our literature review, we suggest that several possible explanations exist for the mechanism for this association. Stasis, hypercoagulability, and endothelial dysfunction, known as the Virchow triad, are believed to contribute to thrombosis.^[20]^ Heavy alcohol consumption may be an independent risk factor for endothelial dysfunction.^[21]^ One of the mechanisms by which alcohol induces endothelial dysfunction is through the nitric oxide (NO) pathway. Chronic alcohol consumption interferes with NO production or release from endothelial cells^[22]^; specifically, high concentrations of alcohol reduce NO synthesis and endothelial proliferation.^[23]^ Furthermore, the proapoptotic caspase pathway is activated by high concentrations of ethanol.^[24]^ Later, alcohol withdrawal can also induce endothelial dysfunction.^[25,26]^ In short, binge alcohol consumption can result in endothelial dysfunction, which leads to increased VTE formation.

Alcohol consumption could also influence coagulation, fibrinolysis, and platelet activities. Heavy alcohol consumption appears to be associated with a higher procoagulant state and an impaired fibrinolytic potential,^[27,28]^ activities which can predispose individuals to thrombosis.^[29,30]^ Acute ingestion of a relatively large but tolerable dose of alcohol transiently enhances thromboxane-mediated platelet activation,^[31]^ and hyperagreeability is observed after acute alcohol consumption.^[32]^ Moreover, long-term alcohol use induces chronic liver disease. Liver dysfunction represents a state of overall decreased liver synthetic function, including a balanced decreased synthesis of the anticoagulant thrombotic factors.^[33]^ In addition, the inflammatory responses are enhanced by both the activation of Kupffer cells and direct metabolic effects of ethanol.^[34]^ Inflammation activates coagulation, and coagulation modulates the inflammatory activity.^[35]^ These alcohol-associated hypercoagulability statuses increase thrombus formation and the risk of VTE.

The strengths of our study are the population-based design, large sample size, and long study period. However, several limitations must be noted. First, the NHIRD does not disclose patients’ socioeconomic status, family history, personal health behaviors (eg, smoking habits or alcohol consumption), laboratory data, or biomarkers. VTE may have been caused by these confounding factors, and they may have influenced our results. Second, the NHIRD only contains records of patients who were admitted to the hospital after being diagnosed with AI; therefore, people with a binge drinking problem who were not admitted to the hospital were unable to be recruited for this study. Third, the severity of both AI and VTE could not be assessed in this study. Fourth, we could not identify either the methods used to diagnose VTE or the accuracy of the attributed ICD-9 code. Previous study revealed as many as 40% of patients identified with VTE-associated ICD-9 codes did not have VTE.^[36]^ Finally, the exact mechanism that connects AI to VTE could not be identified through a retrospective cohort study by using the NHIRD.

## 5 Conclusion

An increased incidence of VTE was observed in the AI group. For people younger than 50 years old, before AI could be an independent risk factor for VTE. Moreover, the incidence of VTE increased with age. These findings provide evidence for physicians that enables them to prevent VTE when managing patients with AI. However, additional studies are warranted to clarify the association between AI and VTE.
